# Notes and News

**Published:** 1902-08-30

**Authors:** 


### Notes and News.

The Duke of Bedford has promised £1,000 a year, for three years, to the Cancer Research Fund.

The annual Congress of German Scientists and Medical Men will be held this year at Carlsbad, commencing September 21st.

The new Queen Victoria wing at the Leicester Infirmary has been opened. It consists of two operating theatres, with all necessary accessory offices and living accommodation for the medical staff, together with offices for the matron and clerk and a ward and rooms for casualty and emergency cases, waiting hall, dispensary, and surgery and examination-room. Messrs. Everard and Pick are the architects, and the scheme has cost about £12,000. Leicester is to be congratulated upon having possession of the services of the late Alderman Lennard, who initiated the collection of the Commemoration Fund, and upon those of the present Mayor, who has before him the complete renovation of the whole of the infirmary.

Lord Northcote has recently opened a hospital for the native poor at Bombay. This hospital inaugurates the second stage of the admirable philanthropic work of Mrs. Adams-Wylie in Bombay. She came to the assistance of the municipality when they were embarassed by the difficulties caused by the drought, and she has now presented Bombay with the fine Adams-Wylie Hospital as a memorial to her late husband. The hospital is complete and well equipped. Its immediate current expenses will be defrayed by the Bombay branch of the Charitable Relief Fund, but after this it will be dependent upon general charity. The hospital has especial claims on the wealthy members of the native community, for it is designed to meet the wants of the sick immigrant poor. The building contains three wards, to accommodate 100 patients, and is supplied with operating theatre, surgery, and all necessary offices. Already Mrs. Adams-Wylie has met with substantial co-operation in her work. The City Improvement Trust granted her a site, Mr. T. Mungaldas has promised an annual subscription and provided a bungalow for convalescents, and other assistance is forthcoming.

A death attributed to the use of a "baby comforter" was recorded recently at Lambeth. The baby died of gastric irritation at St. Thomas's Hospital, the cause being traceable to a dirty "comforter."

Miss F. Blackman has bequeathed £10,000 to Guy's Hospital to found a Blackman ward, £1,000 to the Ramsgate Seaman's Hospital and to the General Infirmary, and £10,000 to the Kent and Canterbury Hospital.

A peeress has resorted to the original idea of receiving the people of the locality in which she is staying, arrayed in her Coronation robes, in aid of the local hospital, a small charge being made for admission to her residence.

The admissions to Westminster Abbey after the Coronation have produced a sum of nearly £5,000. We cannot help regretting that the period for viewing the scene of the Coronation was not extended a little longer for everyone's benefit.

Beriberi has been reported as present on a French vessel which has recently put in at Falmouth. Three sailors succumbed to the disease during the voyage, and four critical cases were landed at Falmouth.

An experiment is being tried by the naval authorities at Malta, where fever is now very prevalent. The fever cases under their jurisdiction are being transferred to the hospital ship *Maine*, which will cruise with the patients in the vicinity of Corfu.

Deaths amongst the troopers landed at Melbourne from the *Drayton Grange* still continue to occur. The commission which is inquiring into the overcrowding of the *Britannic*, the transport conveying troops to New Zealand, is conducting its inquiry privately.

The Maharajah of Gwalior, who has presented £10,000 to the King for the London hospitals, previously showed his munificence towards England by equipping a hospital ship for the use of the British Army during the China Expedition. This scheme cost him £120,000.

The Annual Congress of the Sanitary Institute will be held at Manchester on September 9th. The Secretary of the Congress, J. Reynolds, Esq., will supply intending visitors with a list of hotels and apartments, with the prices charged, if they apply to him at the Municipal School of Technology, Manchester.

The War Office is about to make arrangements for the reception into hospitals at the expense of the Government of invalided soldiers who are likely to benefit by special treatment. During the time of hospital treatment pensions will be withheld. Such men as are cured to the extent of regaining their full wage-earning power will receive no further pension, and the pensions of those who can only partially support themselves will be granted accordingly. The just assessment of this sliding scale of pensions will be an extremely difficult one, and we can imagine that the abandonment of invalid privileges will not meet with universal favour.

